# Long-standing iron-deficiency anemia in an atypical celiac disease – a case report

**Published:** 2014

**Authors:** I Dina, C Iacobescu, C Vrabie, S Omer

**Affiliations:** * Gastroenterology Clinic, St. John Emergency Hospital, Bucharest; ** Histopathology Department, St. John Emergency Hospital, Bucharest; *** University of Medicine and Pharmacy Carol Davila, Bucharest

**Keywords:** anemia, diarrhea, young, gluten intolerance

## Abstract

Celiac disease is a complex disorder characterized by digestive symptoms as well as extraintestinal manifestations, sometimes difficult to diagnose. Commonly described as a disease of childhood, adult celiac disease is a well known entity that should be taken into the differential diagnosis of a chronic diarrhea or of a malabsorption syndrome. The pathogenesis encompasses an autoimmune pathway that acts on a genetic background. The mucosa of the small intestine became damaged in reponse to foods that contain gluten in subjects with genetic susceptibility. The clinical presentation is variable, ranging from typical gastrointestinal symptoms to extradigestive and systemic manifestations. The simple withdrawal of the dietary gluten results in clinical improvement and healing of the intestinal mucosa. We report the case of an young women diagnosed with celiac disease after 7 years of iron deficiency anemia without a clear etiology.

## Introduction

Celiac disease is an immune – mediated disorder of the small intestine with a variable clinical picture induced by gluten ingestion in genetically predisposed subjects [**1**]. The immune response in celiac disease is T-cell mediated because the majority of patients express the HLA-DQ2 and DQ8 molecules [**2**,**3**]. The tissue transglutaminase enzyme has been identified as the leading autoantigen of the disease, making celiac disease a “special” autoimmune disorder, with a well recognized target for the immune response. The transglutaminase binds to the dietary gliadin in enterocytes, inducing immune response and releasing proinflammatory cytokines in lamina propria of the proximal small intestine, followed by an inflammatory reaction characterized by infiltration with chronic inflammatory cells [**4**,**5**,**6**]. Histopathological examination reveals several typical features, according to Marsh classification: intraepithelial lymphocytosis, crypt hyperplasia, villous atrophy [**7**]. The clinical presentation is variable, ranging from a pure „ gastrointestinal disease” with typical symptoms of malabsorbtion like diarrhea and weight loss to nonclassical forms of disease, dominated by extradigestive manifestations – hematologic abnormalities, neurologic or psychiatric syndromes [**7**,**8**,**9**]. There have been described four types of celiac disease, depending on clinical, serological and histological pattern [**6**]. The classic form is characterized by gastrointestinal symptoms of malabsorbtion like diarrhea, steatorrhea, weight loss, flatulence and intestinal villous atrophy. Atypical form exhibit only extradigestive manifestations and histological changes, but not fully villous atrophy. Iron-deficient anemia is the most frequent extraintestinal manifestation of celiac disease, occuring in about 50 % of patients [**10**]. The mechanism of anemia is related to impaired iron absorbtion in the upper part of the small intestine due to villous atrophy of the mucosa [**10**]. The diagnosis of gluten enteropathy is challenging when the disease expresses only atypical extradigestive symptoms. Silent celiac disease encompasses asymptomatic pacients who develop histologic abnormalities after gluten ingestion. The latent form of disease refers to subjects with predisposing HLA-DQ2 and HLA-DQ8 haplotypes, a normal intestinal mucosa on a gluten-containing diet and positive serology [**7**,**11**]. The gold standard of diagnosis remains the biopsy of small intestine associated with a positive response to a gluten-free diet [**1**,**7**]. Gluten withdrawal from the diet should be lifelong because it is not only accompanied by clinical improvement but also by histologic recovery [**1**]. The presence of circulating antibodies-antiendomysium which shows high specificity come along to support the diagnosis.

## Case report

**Fig. 1:**
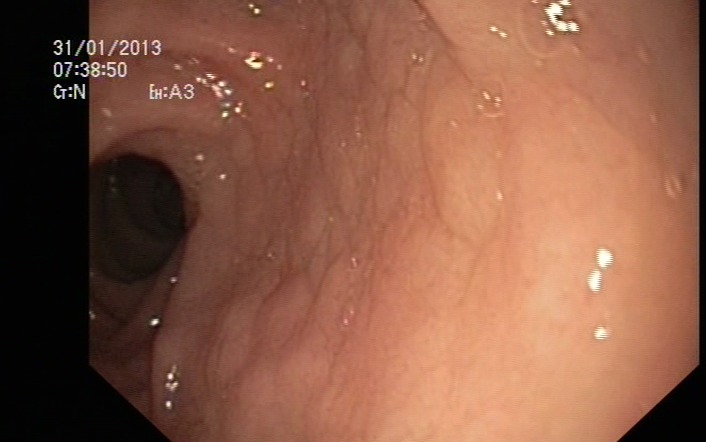
Celiac disease – endoscopic appearance

**Fig. 2:**
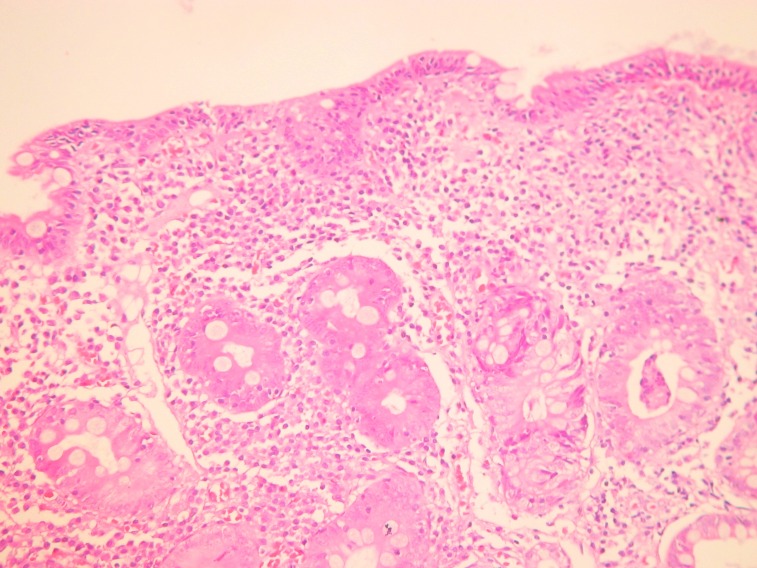
Marked villous atrophy and increased number of intraepithelial lymphocytosis, HE, 200x

**Fig. 3:**
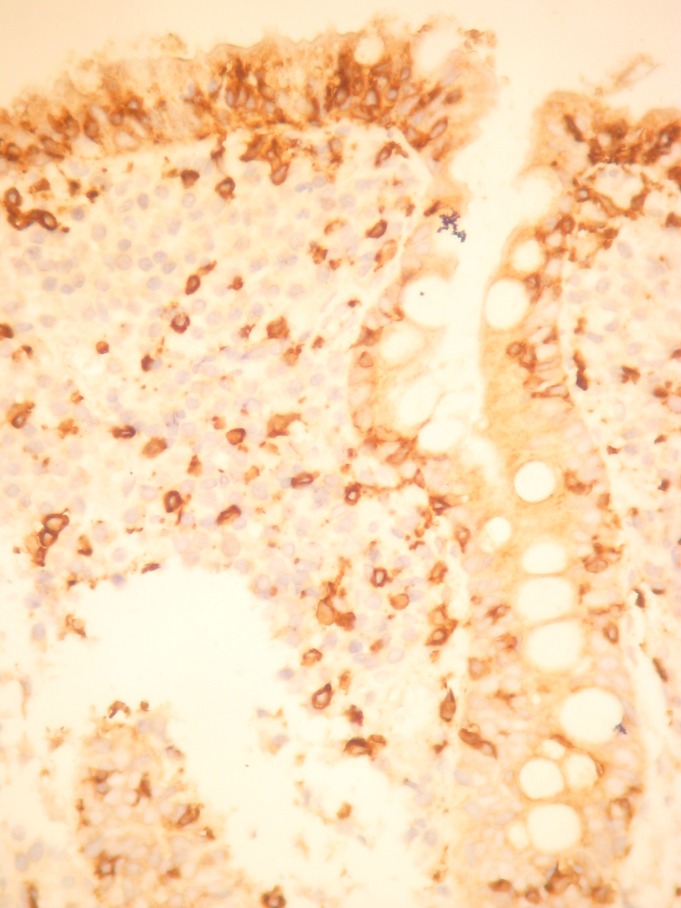
Immunohistochemistry: CD 3 positive intraepithelial T lymphocytes, 400x

**Fig. 4:**
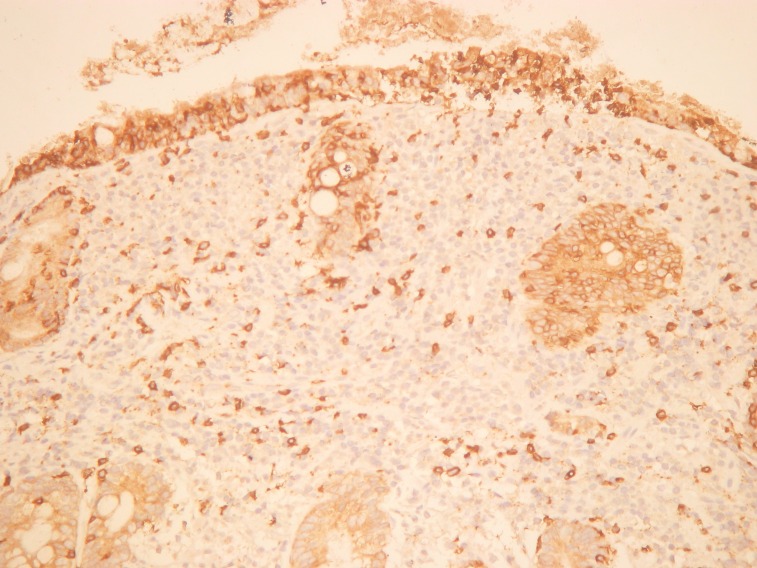
Immunohistochemistry: CD 8 positive intraepithelial T lymphocytes, 200x

A 38-year old female came to our attention with seven years history of mild microcytic anemia, with recent worsening of her general state, for further investigations and a certain diagnosis establishment. Besides the anemic syndrome, treated repeatedly, but inefficient with oral iron therapy, her additional history revealed non-specific gastrointestinal symptoms, „labelled “ as irritable bowel syndrome and managed accordingly. The patient stated for intermittent episodes of diarrhea, without warning signs like pus or blood passage in the stool and apparently without a clear trigger for the abdominal disturbances. Diffuse abdominal pain, flatulence and weight loss completed the clinical picture. At admission to our clinic, the patient complained of weakness, fatigue, diarrhea, meteorism and abdominal cramps. The physical examination showed a ill-looking patient, pale and thin with a lower BMI (17,8 kg/m2). The abdominal examination revealed abdominal distension, without any palpable masses or organomegaly. The laboratory studies showed a moderate microcytic hypochrome anemia with a hemoglobin level of 8,6 g/dl, a normal leukocyte and platelet count. Routine biochemical tests evidenced a mild hypoproteinemia of 5,9 g/dl and hypoalbuminemia of 3,3 g/dl, a low ionized seric calcium of 3,2 mg/dl and a decreased level of circulating iron of 35 µg/dL. The other biochemical results were within normal ranges. The urine examination was normal, without urine protein loss. Viral hepatitis markers as well as HIV antibodies were negative. Coproculture showed no pre absence of germs, the coproparasitological examination excluded the presence of parasites. Complete colonoscopy with terminal ileum visualization reveal no mucosal abnormalities. Upper digestive endoscopy showed the loss of Kerckring folds in the descending duodenum, which is a characteristic feature for celiac disease (**Fig. 1**). Small-bowel biopsies were obtained from the second part of the duodenum and send for histopathologic interpretation. Abdominal ultrasound was performed, with no abnormalities. Based on the endoscopy findings, correlated with clinical and biological data, the diagnosis of celiac disease was strongly suspected and other possible differential diagnosis were ruled out: infectious diarrhea, intestinal parasitoses, inflammatory bowel disease, pancreatic insufficiency, malignancies. Positive serology was also detected and come along to support the diagnosis of celiac disease. IgA endomysial antibody level was 1/320 and the titre of Ig A antibodies directed against deamidated gliadin was elevated, over 142U/ml (normal value <7 U/ml). Histologic examination completed with immunohistochemical tests confirmed the diagnosis of gluten-sensitive enteropathy grade 3 C ( Oberhuber): complete villous atrophy, decreased number of caliciform cells and lymphoplasmocitary infiltration of lamina propria with raised intraepithelial lymphocyte count (**Fig. 2**). No signs of atypical lymphoid infiltrate was detected, excluding a possible superimposed malignancy. Immunohistochemistry examination recognized typical findings: CD3 positive within intraepithelial lymphocytes, CD4 positive in the small lymphocytes of lamina propria and CD8 positive in the small intraepithelial lymphocytes (**Fig. 3**,**4**). The patient was started on a strictly gluten-free diet associated with parenteral iron supplementation, vitamins and minerals substitution. Clinical improvement was noted after a couple of days, with stool normalization and general status recovery. The histologic recovery consequently gluten withdrawal will be assess performing follow-up small-intestine biopsies within 6 to 12 months after starting the the gluten-free diet. The patient was advised to keep life-long the gluten-free diet, in order to achieve a good clinical outcome, regression of mucosal abnormalities and avoid the complications of celiac disease, especially T-cell lymphoma development.

## Discussion

Celiac disease represents an oral intolerance to an ingested protein contained in certain grains, especially in wheat that cause in response small-intestinal mucosal damage immune-mediated [**1**,**2**]. Celiac disease can develop at any age, although originally it was considered to belong to pediatric population. Its general prevalence is increasing, approaching almost 1% in western countries [**7**,**9**,**12**]. Celiac disease may exhibit digestive symptoms, some of them without specificiy, as well as extraintestinal features, making the diagnosis difficult in such circumstances. Iron-deficiency anemia is considered the most frequent laboratory manifestation of celiac disease, independent of the disease type and at the same time the most frequent manifestation of atypical disease [**13**]. A long-standing iron-deficiency anemia was the main symptom encountered in our patient case and played the key role in establishing the diagnosis, raising the suspicion of a possible gluten sensitive enteropathy. Some authors alert for routine screening of celiac disease in patients with iron- deficiency anemia [**12**]. On one hand, at the onset of the disease our patient did not present typical signs of celiac disease, but only biological abnormalities consistent with mild anemia than worsen progressively. On the other hand, gastrointestinal symptoms completed the clinical picture at some point in the course of the disease, but the lack of specificity and the absence of a trigger like certain foods delayed the correct diagnosis. The endoscopic appearance, although not pathognomonic was highly predictive for celiac disease. Finally, the diagnosis was sustained through histologic examination of the biopsy samples took from the distal part of the second duodenum. The well-known microscopical features, typical for celiac disease, were found on biopsy specimens. Ig A antibodies antigliadin and anti endomysium showed high titres, contributing to support the positive diagnosis. Starting a gluten-free diet is the mainstay of treatment and has important consequence not only on the course of the disease, improving the clinical status and inducing total or partial histologic normalization, but also on the extraintestinal manifestations. Withdrawal of gluten includes the obvious sources of gluten, like wheat, oats, rye, barley as well as all the „hidden„ souces that means alimentary products that contain small amounts of gluten [**12**]. Histological recovery of the small bowel mucosa will have positive effects on iron absorbtion in the duodenum and upper jejunum resulting in correction of anemia. In our case, the patient’s anemia significantly reversed after starting the gluten-free diet, cell blood count showing an increased hemoglobin level at 10 g/dl. Histologic normalization of the mucosa will start within 6 to 12 months after taking the gluten-free diet, with incomplete or total recovery of mucosal arhitecture .The patient’s outcome was favourable, with both clinical and biological improvement. The rapid clinical response announced also a favourable long-term prognosis by decreasing the risk of both benign and malignant complications development. The issue of this patient’case is that a mild iron-deficiency anemia in an young women should never be ignored and ascribed maybe to ginecological disorders, but should promptly alarm for further investigations of the digestive tract.
